# Grüt: A Gardening Sensor Kit for Children

**DOI:** 10.3390/s16020231

**Published:** 2016-02-16

**Authors:** Fabrizio Valpreda, Ilaria Zonda

**Affiliations:** 1 DAD—Architecture and Design Department, Politecnico di Torino, Torino 10129, Italy; 2 MediaLAB Amsterdam, Amsterdam 1091 RZ, The Netherlands; ilaria.zonda@gmail.com

**Keywords:** gardening, children, Internet of Things, virtual avatars, the tamagotchi effect, educational

## Abstract

Food waste is one of the main problems in our society. This is mainly caused by people’s behaviors and attitudes, which influence the whole food chain, from production to final consumption. In fact, food is generally perceived as a commodity by adults, who transmit this behavior to children, who in turn do not develop any consciousness about food’s source. One way to reduce the problem seems to be by changing consumers’ attitudes, which develop during the early years of childhood. Research has shown that after attending school garden classes, children’s food-related behavior changes. Growing crops is not always easy—it can’t be done in the domestic space, and this lead to a loss of the long term positive effects. This paper presents a project that tries to teach children how to grow their own food indoors and outdoors, mixing real and virtual reality, connecting something natural like a plant to the Internet of Things (or IOT, a network of physical objects virtually connected to each other and to the web). The use of sensors related to an app makes this process more fun and useful for educational purposes. The aim of the project is to change children’s attitude towards food, increasing their knowledge about production and consumption, in order to reduce waste on a long term basis. The research has been developed in collaboration with Cisco NL and MediaLAB Amsterdam. The user testing has been executed with Dutch children in Amsterdam.

## 1. Introduction

Every single link in the food chain, from production to its end with consumption, shows a significant waste of edible food [1]. This problem is caused by uncertainty, attitude, human behavior, market rules and local/global policies. The project presented in this paper focuses on consumers’ attitudes towards food. The largest amount of waste occurs in the last part of the cycle, where almost 31% of the food is wasted by consumers after buying it [2]. This means that final users have an enormous influence on what happens to the whole food cycle, and the effects of their behavior affect the supply and demand of every product. The urbanization and the globalization of the food industry caused the personal proximity between producer and consumer to disappear, removing the basis of trust in the production process and the quality of food [3]. In time, this changed consumers’ perception of what food really is: they see it as an industrial product, rather than a valuable resource. This shift in attitude towards food means that the consumers are more likely to waste food than people more aware of the value of food production process [4]. Gardening has a positive long term effect on children’s perception and attitude towards food, developing in them a stronger sense of responsibility, respect, care and environmental awareness [5]. This paper proposes a combined system of sensors and an app that have the goal to help children in the process of growth, giving the necessary support and all the information they need through a virtual avatar. The goal of the project is to extend the experience of school gardens also in private houses, creating in children a stronger bond and awareness towards plant life and value as food. The paper is organized as follows: Section 2 explains how the feeling of ownership can influence children’s motivation in taking care of something, shows the importance of school gardens in children’s education and describes the so called “Tamagotchi Effect”. Section 3 explains the project, describing both the sensor system and the app. Section 4 discusses the results of user testing. Section 5 proposes possible future work.

## 2. Background

### 2.1. Children’s Feelings of Ownership

The state of ownership, or psychological ownership, is defined as the state in which the child feels that an object is his [6]. The feeling of ownership reflects the relationship between the child and an object (material or immaterial) in which the child perceives it as being part of his extended self. The extended self is the sum of all items that the child regards as a part of who they are (limbs, parents, friends, pets, *etc.*) [7]. Because of this perceived relationship, the kid tends to care for it in the same way he would take care of his own things. The most powerful means by which a child invests himself in an object is to create it. Creation requires the child to invest his time, energy and even his values and identity. The created object is attached to the child because it is his product, derived from its being and is formed through his efforts [5]. Ownership over an object answers three important human needs: competence (the feeling of being good at something), self-identity and having a place [8]. At the same time, the needs are also factors that contribute to the child’s development of ownership, making it a continuous interaction. Because of this continuous interaction a child reflects on his relationship with the object and develops a sense of responsibility for it. The innate desire of being good at something drives a child to explore and interact with items in his environment. This interaction results in the child exercising control on them and gaining a feeling of competence. Being in control of an object comprises a large part of what means to possess it. The possession expresses child’s ability to exercise control over his environment, in the same way that his limbs give him the ability to control and shape his surroundings. The more control the child can exercise over an object, the more he associates it with himself developing feelings of ownership over it. By experiencing an interactive process with the object, the child learns more about it establishing and maintaining a sense of identity [7]. Ownership can lead to a child taking responsibility for an object. Responsibility is defined as the habit of choosing, and accepting the consequences of the choice of behavior. In other words, when a kid takes ownership over an object, he chooses to accept the consequences of his actions (good care or negligence).

There are a few factors that contribute to the development for a child’s sense of responsibility. He must:

- Be committed to a task;
- Work at tasks persistently to practice the skill needed to complete them;
- Be internally motivated;
- Have sufficient information to know what is expected of him;
- Have the trust and respect of adults around them;
- Receive the guidance and support of adults when needed [9].

### 2.2. School Gardens

In the Netherlands each year 6000 to 7000 children aged 9–10 attend a program at a school garden near their school. The program was developed by Amsterdam’s Centre for Nature and Environmental Education (ANMEC) as part of an initiative to make Nature and Environment classes more practical. The authors interviewed Elena Francissen, a consultant at ANMEC, obtaining the following information: in summer, at the beginning of the school courses, the children receive theoretical classes where they learn the basics of gardening. By the end of the summer they start attending the school gardens for an hour and a half each week. Each child receives a plot of land with pre-cultured plants to take care of. At the end of the school year the children harvest their plants and at some school gardens get to cook dishes with their harvested fruits and vegetables. A study conducted by ANMEC on children’s satisfaction with the school gardening classes, highlighted how many children like the program and what they mostly like about it. The study has shown that 64% of them really like gardening, 21% have no special feeling either way and 11% find it boring; 91% of all the interviewed kids indicated that they have learned something, 7% indicated that they have not learned anything and 2% did not answer. The children who indicated that they find gardening boring were more likely not to have learned anything.

The activities that the children enjoy the most are: gardening (taking care of the plants), harvesting and cooking with the harvested crops. Most children take the care of their plants very seriously, regardless of their prior knowledge. Just because they receive the plants, they develop responsibility for them and this often involves naming the plants or describing the plants as their children’. At the beginning of every trip to the school garden, the children are very excited to check on the progress of their plants. They get annoyed or upset if their plants suffer because of another child’s negligence or if an animal eats their plant and become excited if the plant has done well. Children often are more likely to form a bond with their biggest and/or most beautiful plants. They are less likely to do that with plants that wither or die because they see how well the plants of other children are doing and feel like they have failed. Another point of pride is cooking after the harvest, giving children great satisfaction for the results they achieve. The reward for the effort put in the process by children is felt within the successful growth process and cooking. The interview revealed that after the school garden program, most of the children have an interest in continuing gardening at home, but they are not always able to do that because of lack of room and guidance by their parents [10]. The sense of ownership is developed by the school garden by giving each child at least one plant to take care of, but fails in growing the sense of responsibility, because the kids can take care of their plants only once a week, thus producing a lack of continuity. In the amount of time between each visit to the garden someone from the staff takes care of the plants, thus nullifying the cause-effect relationship that better develops responsibility. In the project described in this paper the authors intend to go beyond the school garden experience, creating a one-to-one close relationship and experience between the child and the plant, where everyday needs are important for the plant’s wellbeing and the child’s education.

### 2.3. The Tamagotchi Effect

The children have one goal while gardening: to make sure their plants grows well and bear fruits. In order to accomplish this task, they need to know how to properly take care of their plants. By providing the child with feedback during the plant’s growth process, the child has more insight into his behavior and knows what he has to change about his actions in order to better take care of it [11]. According to Massey [4], a person’s values are developed before the age of 21. Up to age 7 a child’s mind is like a sponge, absorbing everything around him and accepting much of it as true, especially when it comes from parents. Between the ages of 8 and 13 children start copying other people, including their parents. Instead of blindly accepting the values of others, a child explores and assesses their values, to determine whether he is comfortable with them or not. The reference person can be a parent, a teacher, a friend or, as we are about to explain, a virtual fellow. The most striking example of a virtual companion that influences children a lot is Tamagotchi. It used to be a popular game device in the 90s and children tended to develop feelings of ownership for their Tamagotchi pets (the Tamagotchi effect [12]). The goal of the game is to keep the pet alive and entertained. By giving the user a simple interface and clear goals in short intervals (feeding, bathing, *etc*.) and easy ways to complete these goals, the Tamagotchi manages to improve the rate in which the user is engaged. Tamagotchi gives the user the experience of owning a virtual pet; the pet goes through several stages of growth and will develop depending on the care the user provides. The user needs to feed, clean and take care of the Tamagotchi during its life span. If the user takes good care of the pet, it will be smarter, happier and require less attention as an adult. Should the user not take proper care of the pet, its condition would deteriorate and eventually die. Tamagotchi were originally designed for teenage girls to give them an idea of what it would be like to take care of a child [13]. However, the Tamagotchi quickly become popular under people of all ages and as of 2010 76 million units had been sold worldwide. The Tamagotchi’s rise in popularity is due to its ability to fulfill people’s innate desire to take care of something or someone [14] and it has a simple gameplay formula: a mobile experience that the user engages with in several short sessions a day [15]. We can explain the popularity of the Tamagotchi in the 90s with its successful design allowing children to take ownership over their digital pet. It does so by making use of all the factors that influence the development of ownership and it is designed in such a way that the amount of errors the user can make is limited. Tamagotchi allows the child to control the creation of the pet: he can pick what kind of pet he wants and give it a name. The Tamagotchi also limits the amount of mistakes the child can make in the growth of the pet. It clearly states beforehand what the needs of the pet are, when the pet needs the child’s attention and how he can meet them. This gives the child the feeling that he has control over his pet’s growth, which in turn makes him want to spend more time with it, taking responsibility for its well-being and forming a bond with it. The Tamagotchi effect is described as the development of an emotional attachment with both material (machines) and artificial beings (avatars) that otherwise do not have any real emotions [16]. An avatar is an artificial (digital) companion that is an intelligent and cognitive representation of a character, person or object. When a child is asked to care for an avatar he will feel an emotional connection to the avatar and experience it as intelligent, if the child’s actions have an influence on the avatar’s condition and if the avatar offers feedback on the child’s actions [16].

The system can provide its feedback in form of emotions the child can interpret to determine the conditions of the Tamagotchi. The emotions expressed can be as simple as a symbol that represents a specific emotion: a heart for happiness or a tear for sadness [17]. For the child to perceive the avatar’s emotions as real, it must be based on an internal value system that is adaptive to the avatar’s conditions [18]. In the case of the Tamagotchi this was an algorithm programmed into the device that dictated when the pet was hungry or needed to be cleaned.

## 3. GRÜT

### 3.1. Ownership and Responsibility

The system developed by the authors tries to respond to the specific needs required by the child to develop a relationship with his plant, the most relevant condition to learn why and how to take care of it. As stated in Section 2.1, the sense of ownership is achieved when the feeling of competence, identity and having a place are achieved. To give the child the technical skills and insights, in order to be able to succeed in the growth process, is the first step to make him feel competent. The direct connection between the child and the avatar leads the child to identify the avatar as a close fellow, to which the kid can relate and deal with. The possibility to customize the virtual environment in order to reproduce the reality in which the child lives and experiences, gives answers to the need of having a place, in the sense of feeling comfortable, in a real and virtual setting. 

Giving daily tasks strengthens the feeling of responsibility. The child is then asked to perform certain actions every day, in order to keep the plant healthy and the virtual avatar happy. The direct perception of how much the child’s actions influence the wellbeing of the plant motivates him in being steady in performing the daily tasks every day. The presence of a virtual avatar, finally, gives the child a guide, a teacher and a friend, to whom he can relate for insights in the results of caring for the avatar’s (and plant’s) wellbeing.

### 3.2. Applying the Tamagotchi Effect

**Figure 1:**
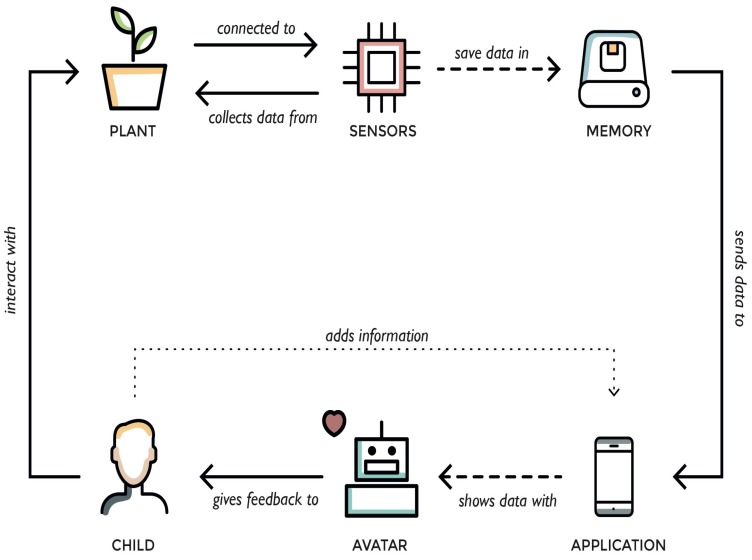
Technical flow.

A physical plant, just like a Tamagotchi, contains a set of basic factors that determine its growth condition. These factors are: the amount of sunlight it receives, the humidity of the soil and the temperature of the environment [19]. The condition of the plant can be made transparent by measuring these three factors with suitable sensors. The sensors are attached to the soil around the plant and can send the data to a mobile app; the device will send the data to the cloud and comparing it with the values that are preferred for optimal growth of the plant. Depending on the amount of light, water or heat/cold the plant receives, the avatar will display the proper emotion reproducing the plant’s condition (Figure 1). By using the Tamagotchi effect as gamification element, the digital application can present the data that is gathered in a way that the child is accustomed to (through a game character), and finds fun and interesting. This process will encourage the child to engage more with it. Since a physical plant also has needs that determine how well it will grow and as these needs can be transferred to the digital realm, it can be represented in a digital application as an avatar.

### 3.3. Concept

The first sensor prototype was built using an Arduino Yun, with three sensors (light, temperature, moisture) attached to it. The sensors detect the data and store them in the Arduino board, that sends them via Wi-Fi to a database and a web App. The Arduino needs to be connected to a computer to set up the Wi-Fi connection and connect the sensors. The whole system needs to be powered either with a USB cable connected to the laptop or with a solar panel, which we choose to keep the prototype easy to move. A red LED light would turn on when wrong data was collected from the sensors to notify the child to check on the website what was wrong.

**Figure 2:**
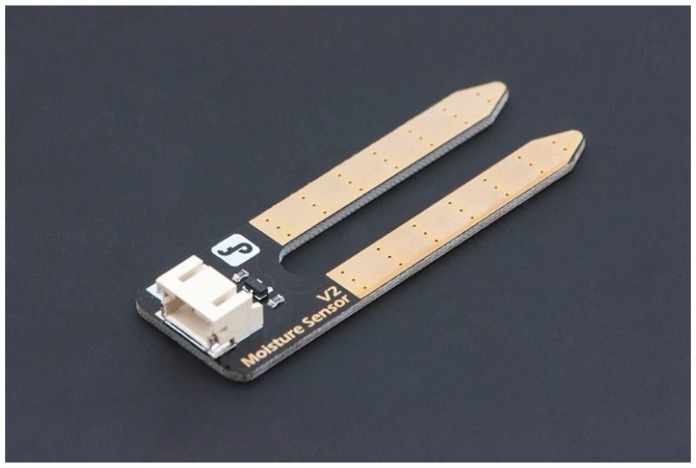
DFRobot Soil Moisture Sensor (Arduino Compatible) Immersion Gold.

**Figure 3:**
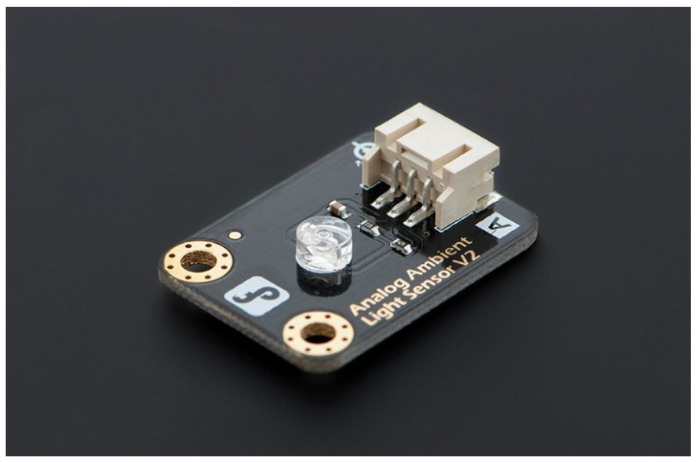
DFRobot Analog Ambient Light Sensor.

**Figure 4:**
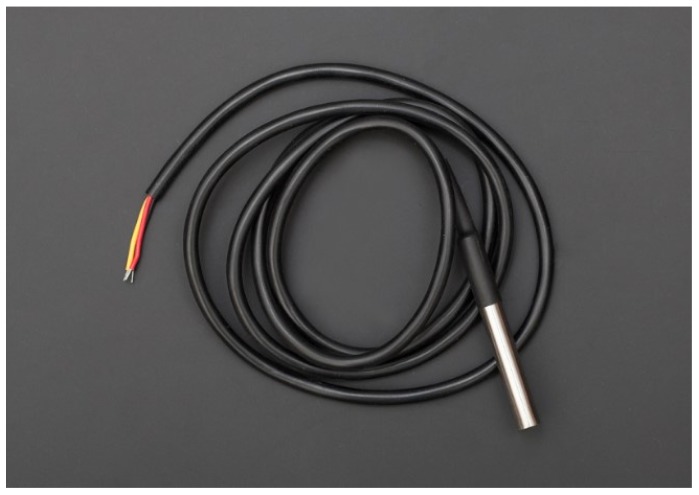
DFRobot Waterproof DS18B20 Digital Temperature Sensor.

List of components:

- Arduino Yun;
- DFRobot IO Expansion Shield for Arduino V7.1;
- DFRobot Soil Moisture Sensor (Arduino Compatible) Immersion Gold (Figure 2);
- DFRobot Analog Ambient Light Sensor (Figure 3);
- DFRobot Waterproof DS18B20 Digital Temperature Sensor (Figure 4);
- DFRobot Terminal Sensor Adapter V2.0 (for temperature sensor);
- Female headers to connect Expansion Shield to Arduino Yun;
- Red LED;
- 270 or 330 Ohms resistor;
- Solar panel that can charge the Arduino;
- Micro USB cable (to connect Arduino to power source).

The first tests gave promising results, with the platform showing an affordable and effective layout. However, it turned out very soon that such prototype would have led to final product exceeding the performance needed in the specific context of use: it would also have been too expensive and uselessly sophisticated as it would be in close contact with children, water and dirt. For these reason a much lighter design layout was chosen, in order to keep costs and complexity commensurate to the children use.

The final prototype consists of a mobile app and a sensor kit. The sensor kit is built upon three sensors, each one capturing temperature, humidity and light data. The child puts the kit in the soil next to the seedling, then the sensors will send data about the plant’s condition to the app, which provides the child with an overview of the plant’s condition and tells what is needed in order to take proper care of it needs. The primary target children are about ages of 9 to 10, who have an interest in gardening but have almost no experience. The secondary target are parents who want to involve their children in gardening, but who have no time and/or knowledge to properly teach them about it.

### 3.4. The Sensors

**Figure 5:**
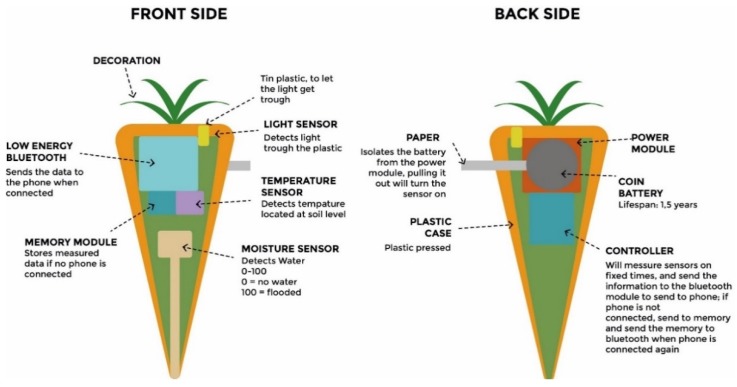
Sensors’ technical specifications.

The sensors (light, temperature and humidity) are packed inside a plastic case, which can be customized in its external shape. The case contains also a coin battery and a controller that checks sensor activity and received data on a fixed time cycle, sending them to the Bluetooth module, which provides data delivery to the mobile device. If the phone is not connected, the data are stored in a small memory, ready to be sent during the first subsequent connection(Figure 5).

### 3.5. The App

The application is designed to provide the child with insights about the growth process and the health status of his seedling, an easy checklist of activities to be performed and, most of all, the avatar must automatically react to the kid’s interaction with the physical plant. When the application is launched for the first time, a small tutorial immediately runs, explaining each graphical user interface element.

**Figure 6:**
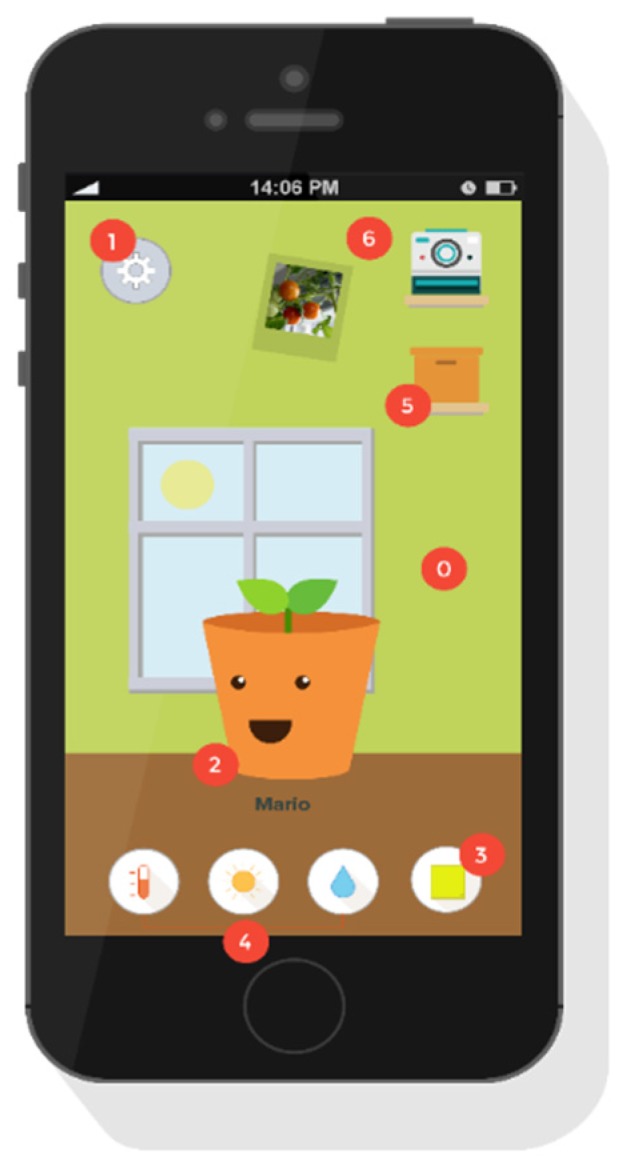
App interface.

Here is a short overview of the app’s contents (Figure 6):

0. Environment

Everything on screen is customizable (moveable) and interactive. The light in the room and the weather displayed outside, change according to the time of day and the real climatic conditions. The child can recreate the environment of his own house, where the real plant is.

1. Menu 

All the system functions are hidden by the menu. When the child opens it, he can edit his personal profile, the room/garden currently on screen and set up/change system settings.

2. Selected Plant 

The avatar displays the attitude/ mood of the plant through facial expressions and text bubbles. If the child clicks on the avatar he will be asked to input certain data related to the physical conditions of the plant (amount of leaves, status of the soil and other observations). This data will be used to further determine what actions appear in the to-do list, as well as the appearance of the plant and what it will tell and ask the child to do. For example, if a plant has moldy soil and some brown leaves, the checklist will add the following item “trim brown leaves with scissors”. The avatar will appear with some brown leaves. By clicking on it, the plant will also explain how mold affects it, how it’s formed and how it can be prevented. Once the leaves are trimmed, and the mold removed, the child has to input the updated information and the avatar will change its appearance to a happier mood. If the child takes proper care of the plant and gives it attention (by going through the checklist every day and answering the virtual plant’s questions) the avatar will be happy and have a positive attitude towards the child, thanking him for his efforts. If not, the avatar will start getting grumpier and get a negative attitude. The changes in the avatar happen immediately after the inputs, to display the strict relation between actions and consequences.

As the real plant grows, the avatar will grow accordingly. A small plant is portrayed as a baby pot, and the bigger the plant gets, the bigger the avatar gets, to the point that it will start getting flowers and fruit. The plant will also tell the child stories with educational purposes. These stories are told by the plant, shown in animations in a text bubble above the plant’s head and cover topics that are related to the plant’s growth.

The stories are kept intentionally short, so that the information can be dosed out into small bits and the child won’t get bored or it won’t lose attention. As time passes the plant will go more in depth, so that the plant will educate the child about its needs helping the child to better understand how the plant grows and why it behaves the way it does.

3. To Do List 

The to-do list gives an overview of all the actions the child must complete on a given day. It is organized into small daily tasks and longer term big tasks. The daily tasks consist of basic assignments that the child can do every day, for example, ensuring the plant has enough light and water, temperature and listening to the plant’s daily conditions. These duties can automatically be checked off by the application: in fact, the app can track the light water and temperature, through the sensors. Big tasks can be checked off by the child after he has performed them. By tracking the to-do list, the application can also tell whether the list item can be checked off or not. A question mark is located at the end of the list of items, and the child can click those, if he needs more information about how to perform them. When clicked, the plant will give a fast and simple explanation about the specific task. If the child does not perform a task, the application will record the missing action. Then, the more the child neglects the plant, the grumpier it will get. The more the child keeps track of the plant’s progress and performs tasks in the to-do list, the happier the plant will be, and the more will show affection towards his owner. 

4. Status Stickers 

The status stickers are meant to provide a quick overview of the plant, possibly receiving too much or too little light, water or living in wrong environmental temperature conditions. When the plant is added to the room, its status will be shown on the stickers under the bookcase and a to-do list will be generated based on data gathered by sensors and the child’s observations. The plant also displays his status through its mood (expressions & text bubbles).

5. The Box

The cardboard box adds other features to the app: the encyclopedia and the recipes book. The first is the knowledge base where children and parents can find all the information they need about plant’s growth, displayed through videos and illustrations, to be easy to understand and fun to play.

The recipes book shows recipes that are related to the plants that the child is growing, giving insights for a conscious consumption. 

6. Polaroids 

The child can add pictures to the wall by clicking on the Polaroid camera. It is also possible to drag the Polaroids. This allows the child to make the interface better fit their needs. It will also be possible to share the pictures online, uploading them on social networks.

## 4. User Testing

**Figure 7:**
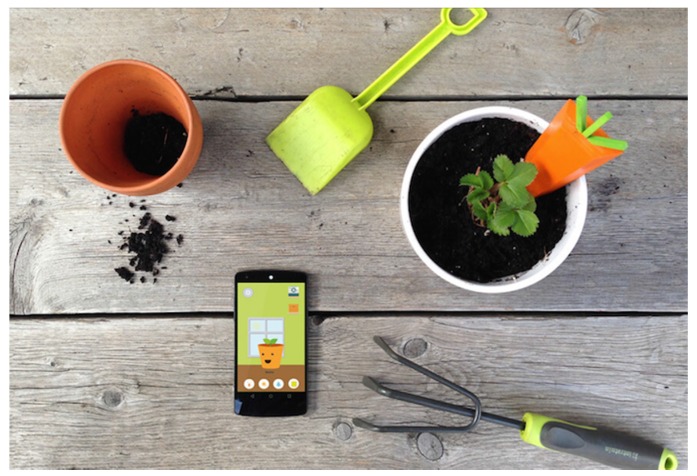
The sensor mockup and the app.

The first clickable prototype product was tested with four children, aged 9–10, and their parents. Two families were involved in the tests: the first family consisted of mother, father, and three children, aged 9–10. The family lives in Ypenburg, a suburb of The Hague in a nice house with a garden in the backyard. The three brothers, already take care of their plants in the garden, and each of them have their own to look after. The second family consisted of a single mother and her 9 year old son. They live in an apartment in The Hague, near the city centre, without a garden. Their house has a small balcony outside where they grow a few potted plants. To test the app the team used a potted plant, with a mockup sensor (Figure 7) attached to it and an iPad with the application prototype on it as visual aids to explain the concept and how the app worked. The novelty of the idea intrigued the tester kids from the beginning, because they were able to make a connection with a real plant the same way they would with a real/digital animal. After explanation, the children and parents received a list of task to complete and questions to answer. Based on the ease of which they completed the task and where they struggled, the strong and weak points of the interface were determined.

The tasks and questions were:

- Add a plant to the garden;
- Determine the mood of the plant;
- Explain what the elements on the screen are;
- Determine the condition of the plant;
- Does the plant have enough water?
- Is the plant receiving enough sunlight?
- Is the plant at the right temperature?
- Do you know how to properly care for a plant?
- If this plant were yours, would you take care of it?
- How often would you check on your plant?

**Figure 8:**
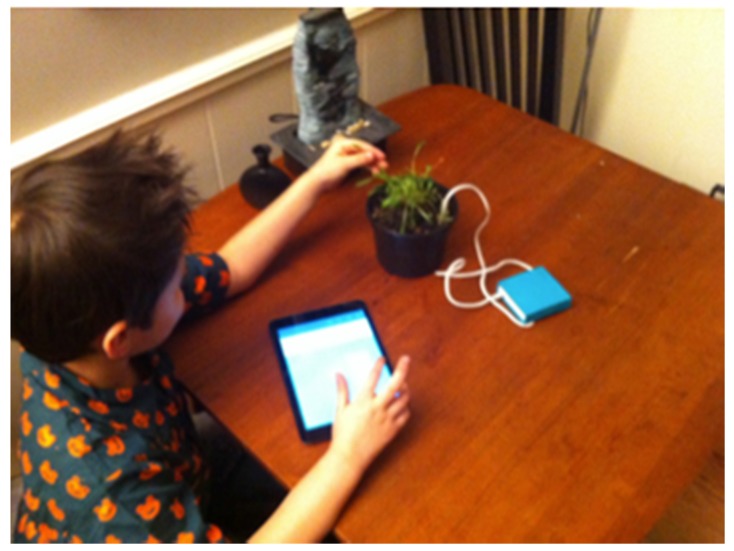
User testing.

Adding a plant to the garden went smoothly, since they did not encounter any problem with the form, they immediately referred to the avatar to confirm the status of the plant (it is the first thing they notice when they open the application), and the intentions of the buttons were clear (Figure 8).

Problems were easily solved by themselves. When the avatar stated that it was too warm, they put the plant far from the heater so it received less warmth; their first reaction was not to refer to the status buttons for help. They automatically started clicking around the interface to see what was interactive and what was not—this way was helpful for them to discover the status pop-ups. The status pop-up was clear in its purpose: to provide extra help if and when needed.

The prototype tested at the beginning differed from the final one by having only the status stickers, which showed the situation of light, temperature and humidity. The avatar was designed to show a limited variety of emotions only: very happy, normal, sad, dead. All of the children reacted very positively to the avatar and could easily interpret the state of the plant based on the mood the avatar expressed. However, a longer user test has revealed that the avatar was getting “boring” after a few days of use due to the lack of complexity in its set of emotions. The avatar was very monotone and this problem makes the interactions that the child has with his plant less meaningful to him. Specifically regarding the interface design, they found it very functional but not really fun, in the long term.

After the first user test, new functionalities were added (avatar inputs, Polaroids, editable background, to do list, *etc.*) and the application was tested again with the same children, who responded very positively towards the redesign. They found the first design more functional and they liked that the new redesign maintained the functionality, but they were more attracted by the new one because it was more playful. They liked how the checklist and the avatar influenced each other. One of the children found it useful to have a checklist with activities he could do daily, because he would often forget or would not know what he had to do. And he also liked that the plant (avatar) would explain things to him if he didn’t know what to do. The parents liked how educational the application is, without looking like a “homework assignment”. The tasks on the to do list were pretty simple and they felt that their children would be able to do them on their own for the most part, especially since they got help from the app. They also felt that the simplicity of the application was a plus; children didn’t have to enter a lot of difficult data and the items on the checklist were simple and straightforward. The children felt that giving the plant some personality would really help in giving them the motivation to care for it. Parents also liked that the application had a built-it encyclopedia, with videos and texts. They felt it was a handy functionality to have, considering they didn’t know much about growing plants. They also said that by simulating the entire growth of the plant, while it happens in the reality, children can follow the whole process more directly than they usually do in school gardens, where they are involved only during few steps and for a short time.

While developing the idea we were afraid that after taking care of a plant for so long kids could have issues with “eating their friend”, but, by appropriately explaining the lifecycle of the plant from the beginning, children seems to understand that eating “their friend” does not mean killing him but saving him from being wasted, as should also be for every food they could buy from the supermarket.

## 5. Future Work

While the clickable prototype has made it possible to generate many insights, it also has its limitations. The application could not be tested during the whole growth process of a plant because of the huge amount of time needed, the avatar could not display more than a few states and the checklist could not be tested to its full extent because it’s still to be designed. This did make it difficult to gain insight into what problems and opportunities would arise if the child uses the application for longer periods of time. 

In order to gain more insight into how the application will influence the development of ownership in the long term, a fully functioning application must be built in the next iterations of the products. Another step will be the reintroduction of the first layout, based on an Arduino board—this because it will offer a more flexible, powerful and complete set of functions, giving the opportunity to program the behavior of the app depending on the target (adult, child, self-made home gardeners, *etc.*) and a larger variety of plants. Plus, it could be capable of managing wider amount of data, organizing them for more effective purposes, even in a commercial environment. Along with the sensors a proper case to contain them has to be design, to avoid behaviors that could damage the board, even when used by children.

### 5.1. Technical Implementation

The priority must be to technically implement the clickable prototype. This would make it possible to test the application during a whole growth cycle of a plant and in turn would provide insight into weaknesses and opportunities for the application.

### 5.2. Sensors

In order to support a large number of plants, the application must be developed with data on the requirements needed for each plant to grow properly. Market offers many commercial sensors that can be used for that purpose, and they already have libraries with the needs for specific species. This kind of information is necessary to improve the educational aspect of the product, giving the child different results and instructions strictly related to the type of plants they are growing.

### 5.3. Food Waste 

The next iterations of the prototype must also explore opportunities to influence the child to waste less food during the whole lifecycle (production, consumption, recycling). The goal is to give children options for the use of every part of their plant (seeds for replanting, leftovers for composting, *etc.*) and teach them to choose to grow particular plants strictly connected to seasons and geographical area.

### 5.4. Reward Systems 

The concept as a whole can also benefit by implementing a reward systems. By having tiers in the different plants that children can own, it is possible to ease them into gardening by giving them easy plants that grow quickly (like, for example, strawberries) first and later on, as they get better and better, they are rewarded by unlocking higher level plants that require more care, patience and more important skills. This concept of rewarding can also be applied in the editable and customizable background: the more you take good care of your plant, the more you can change the virtual room, color of the pot, *etc*.

### 5.5. Social Networking

The app can create a network of children growing plants, encouraging them to share their products, meet in the real life and interact. These will improve their social interactions and will teach them to manage their output, instead of wasting them. 

## 6. Conclusions

Many other sensors and monitoring systems for plants already exist in the commercial field, but they are mostly used for big crops and cultivations, providing very technical data and graphs. The innovative aspect in Grüt project is the educational insights, the emotional involvement provided by the virtual avatar and the simplicity of the data, all of them easier and more fun for kids to understand.
